# MetaGT: A pipeline for *de novo* assembly of metatranscriptomes with the aid of metagenomic data

**DOI:** 10.3389/fmicb.2022.981458

**Published:** 2022-10-28

**Authors:** Daria Shafranskaya, Varsha Kale, Rob Finn, Alla L. Lapidus, Anton Korobeynikov, Andrey D. Prjibelski

**Affiliations:** ^1^Center for Algorithmic Biotechnology, Saint Petersburg State University, Saint Petersburg, Russia; ^2^European Molecular Biology Laboratory, European Bioinformatics Institute (EMBL-EBI), Wellcome Genome Campus, Cambridge, United Kingdom; ^3^Department of Computer Science, University of Helsinki, Helsinki, Finland

**Keywords:** metatranscriptomics, metagenomics, RNA-Seq, *de novo* assembly, computational pipeline

## Abstract

While metagenome sequencing may provide insights on the genome sequences and composition of microbial communities, metatranscriptome analysis can be useful for studying the functional activity of a microbiome. RNA-Seq data provides the possibility to determine active genes in the community and how their expression levels depend on external conditions. Although the field of metatranscriptomics is relatively young, the number of projects related to metatranscriptome analysis increases every year and the scope of its applications expands. However, there are several problems that complicate metatranscriptome analysis: complexity of microbial communities, wide dynamic range of transcriptome expression and importantly, the lack of high-quality computational methods for assembling meta-RNA sequencing data. These factors deteriorate the contiguity and completeness of metatranscriptome assemblies, therefore affecting further downstream analysis.

Here we present MetaGT, a pipeline for *de novo* assembly of metatranscriptomes, which is based on the idea of combining both metatranscriptomic and metagenomic data sequenced from the same sample. MetaGT assembles metatranscriptomic contigs and fills in missing regions based on their alignments to metagenome assembly. This approach allows to overcome described complexities and obtain complete RNA sequences, and additionally estimate their abundances. Using various publicly available real and simulated datasets, we demonstrate that MetaGT yields significant improvement in coverage and completeness of metatranscriptome assemblies compared to existing methods that do not exploit metagenomic data. The pipeline is implemented in NextFlow and is freely available from https://github.com/ablab/metaGT.

## Introduction

Metagenome sequencing gained noticeable popularity in the past decade, as multiple projects shed light on microbial communities in various ecosystems (Poretsky et al., 2005; Nowinski et al., 2019) and eukaryotic microbiomes (Turnbaugh et al., 2007; Arumugam et al., 2011; Lloyd-Price et al., 2019). However, these studies required the development of novel software tools, as the previously designed methods for conventional sequencing data analysis appeared to be underperforming on large and complex metagenomic datasets. Thus, multiple tools, such as *de novo* assemblers (Li et al., 2015; Nurk et al., 2017), sequence binners (Uritskiy et al., 2018; Kang et al., 2019; Nissen et al., 2021), annotation pipelines (Seemann, 2014, Keegan et al., 2016) and various pipelines for metagenomic downstream analysis (Caporaso et al., 2010; Mitchell et al., 2020) were developed in the past years.

Although metagenomic sequencing may provide insights on species abundances and gene content, it does not show which members of the community and which genes are active, and how this activity depends on external conditions. To analyze gene expression in the microbial community researchers perform RNA-Seq experiments, which may include sequencing of samples under different conditions, time series, as well as complementary metagenomic and metatranscriptomic sequencing.

As complete genomes of the organisms in the community of interest are often unknown, both metagenomics and metatranscriptomics studies heavily rely on *de novo* sequence assembly. While assembly of metagenomes is typically performed with community-established tools, such as MEGA-HIT (Li et al., 2015) and metaSPAdes (Nurk et al., 2017), metatranscriptome assembly software remains at an early stage and no pipeline is currently regarded as a golden standard (Shakya et al., 2019). Among available tools one can name IDBA-MT (Leung et al., 2013) and its derivative version IDBA-MTP (Leung et al., 2015), which utilizes a database of known proteins to reconstruct complete transcript sequences. Another tool, TAG (Ye and Tang, 2016), exploits the fact that metatranscriptomes are often sequenced along with the metagenomic data from the same sample. TAG maps RNA-Seq reads onto a metagenome assembly graph using and further restores paths corresponding to transcripts. Unfortunately, all listed tools appear to be unmaintained for several years and challenging to run under modern environments. Thus, some of the current studies exploit conventional RNA-Seq assemblers, such as Trinity (Grabherr et al., 2011) and rnaSPAdes (Bushmanova et al., 2019), performance of which remains under-examined on metatranscriptomic data.

In this work we present MetaGT, a user-friendly pipeline for *de novo* assembly of metatranscriptomes, which follows the concept of TAG assembler by simultaneous usage of both metagenomic and metatranscriptomic sequencing data obtained from the same sample. We demonstrate that using metagenomic data greatly improves completeness of assembled transcripts compared to sequences assembled solely from metatranscriptomic data.

## Materials and methods

### Pipeline overview

**Figure 1:**
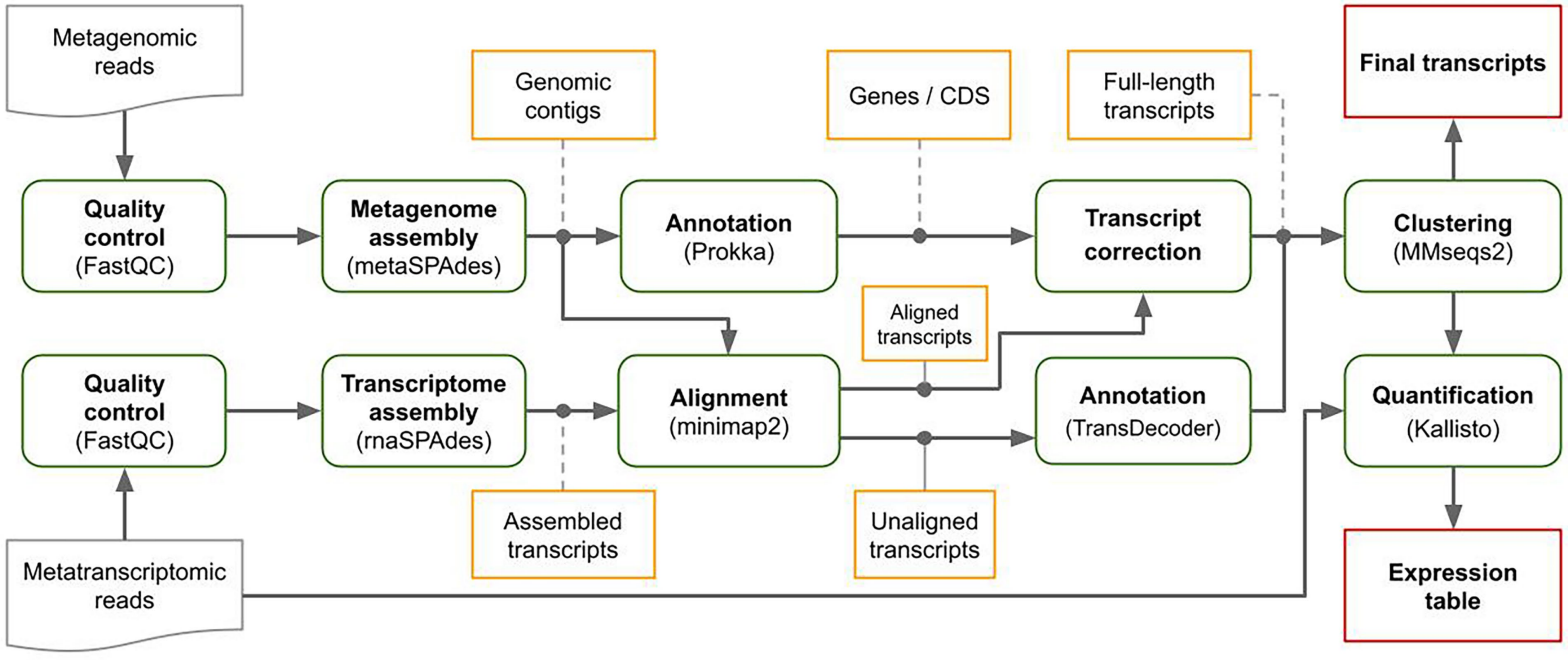
MetaGT pipeline.

MetaGT is a pipeline for *de novo* assembly of metatranscriptomes, which is based on the idea of combining both metatranscriptomic and metagenomic data sequenced from the same sample. First, MetaGT pipeline assembles metatranscriptomic and metagenomic reads individually with rnaSPAdes (Bushmanova et al., 2019) and metaSPAdes (Nurk et al., 2017) respectively. Metagenomic contigs are then annotated with Prokka pipeline (Seemann, 2014). Alternatively, a user may provide assemblies and the annotation obtained with software of their choice. Further, MetaGT aligns transcriptomic contigs to the genomic fragments with minimap2 (Li, 2018). These alignments are used to extend, merge and correct assembled transcriptomic contigs into full-length transcripts. Optionally, unaligned contigs are annotated with Transdecoder^1^ and clustered with previously obtained full-length transcripts with MMseqs2 (Steinegger and Söding, 2017) in order to avoid duplications. Finally, the resulting set of transcripts is quantified using Kallisto (Bray et al., 2016). The scheme of the pipeline is presented in Figure 1.

### Transcript correction

**Figure 2:**
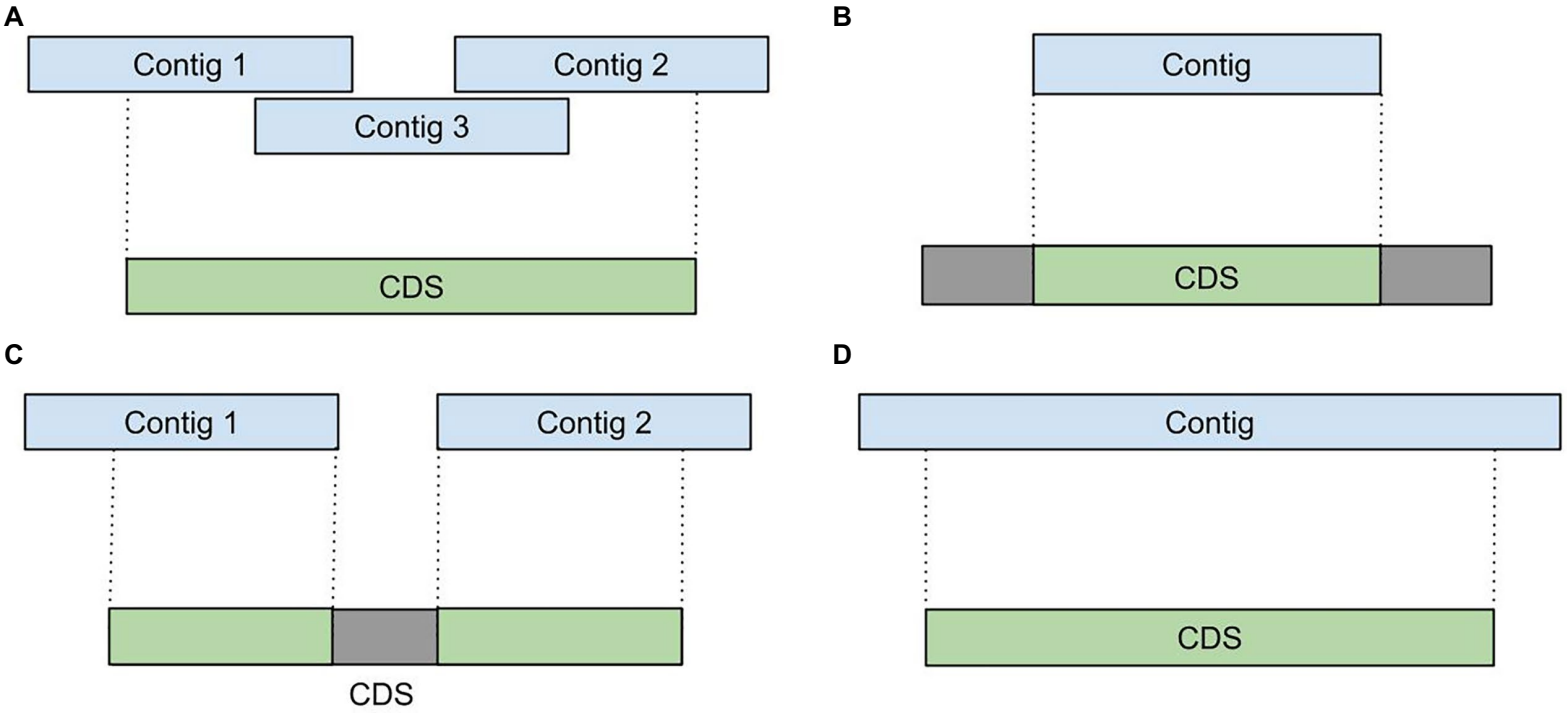
Assembly artifacts typical for metatranscriptome *de novo* assembly. **(A)** Fragmented transcripts. **(B)** Partially assembled transcripts. **(C)** Fragmented and partially assembled transcripts. **(D)** Transcripts containing intergenic sequences.

Due to extremely uneven coverage depth and presence of homologous genes in diverse microbial communities, assemblers tend to generate incomplete and fragmented transcript sequences. For example, during the analysis of various metatranscriptomic samples we detected such assembly artifacts as: (i) fragmented or overlapping transcripts (Figure 2A), (ii) incompletely assembled transcripts (Figures 2B,C) and (iii) extended transcripts containing intergenic sequences (Figure 2D). To improve a metatranscriptome assembly MetaGT implements a procedure that corrects assembled transcripts based on their alignments to the annotated metagenomic contigs.

First, coordinates of the aligned transcripts are compared against positions of the сoding regions predicted by Prokka in the metagenomic contigs. This allows to identify incompletely assembled and fragmented transcripts. For further processing MetaGT selects only coding regions that have at least 50% of their bases covered by the assembled transcripts. This threshold is merely a default parameter and can be modified by the user.

Further, MetaGT merges selected coding regions with the respective assembled sequences by concatenating fragmented transcripts and filling in missing sequences. Moreover, MetaGT removes excessive fragments that map outside of coding regions. Thus, transcriptomic contigs representing operons with several coding regions are split into individual sequences. During the consensus step MetaGT prioritizes assembled sequences in order to preserve any variants and modifications detected in RNA-Seq data. The output of this procedure is a set of transcripts, in which each of them includes a single complete coding region.

### Analysis of unaligned transcripts

Metatranscriptome sequencing may also capture mRNAs that are not produced by the microbial community itself, for example, products of food from gut microbiome samples. Since assembled sequences corresponding to such mRNAs are unlikely to map to the metagenomic contigs, they are processed separately. For all unaligned sequences MetaGT uses TransDecoder^2^ to identify reliable transcript candidates. To avoid duplicated transcripts in its output, MetaGT further uses MMSeqs2 (Steinegger and Söding, 2017) to cluster transcripts reported by TransDecoder with full-length transcripts obtained *via* correction step. If a user wishes to exclude unaligned sequences from the analysis, this step can be turned off *via* command line options. The resulting full-length transcript sequences are saved to a FASTA file, in which transcripts are marked as aligned or unaligned.

### Datasets used for testing

To test MetaGT we simulated metagenomic and metatranscriptomic data based on 3 sets of genomes with various complexity: (i) Simple7: 7 bacterial genomes, (ii) Medium20: 20 bacterial genomes, and (iii) Complex32: 32 bacterial genomes, some of which belong to closely related strains (Table 1). The composition of the simulated data was selected in order to test the applicability of the developed software to bacterial communities with different properties. Indeed, these obtained sets of genomes are synthetic and do not reflect any actual bacterial communities known to date.

**Table 1:** Datasets used in this work.

Dataset	Data type	Genomes	Community complexity	DNA reads	RNA reads
Simple7	Simulation	7	two members of the genus *Corynebacterium*	5 M	10 M
Medium20	Simulation	20	two members of the genus *Corynebacterium*	5.8 M	10 M
Complex32	Simulation	32	2 strains *Escherichia coli*; 3 members of the genus *Shigella*; 2 members of the *Salmonella*; 4 members of the *Lactobacillus*; 2 members of the *Corynebacterium*; 2 members of the *Desulfosporosinus*	7.3 M	10 M
Mock16	Synthetic community	16	2 members of the genus *Klebsiella*	6.3 M	7.2 M
HumanGut	Human gut microbiome			6.8 M	8.7 M
SnailGut	Gut microbiome of deep-sea snail			34 M	23 M

Mock16 is a real sequencing dataset obtained from an artificial mix of 16 distinct bacteria. HumanGut is a real dataset sequenced from a gut microbiome. SnailGut is a real dataset of gut microbiome of a deep-sea hydrothermal vent snail.Simple 7, Medium 20 and Complex 32 are 3 simulated datasets generated based on artificial mixes of bacterial genomes.

During the simulation each bacteria was assigned a random relative abundance value, such that the abundance distribution within the simulated community resembles a distribution of the real one. These values were used to simulate metagenomic Illumina reads with InSilicoSeq software (Gourlé et al., 2019). Further, each gene was assigned an arbitrary expression value in a similar manner: artificial gene expression patterns should be similar to ones observed in real-life data. To obtain the resulting gene abundances for simulation, generated expression levels were multiplied by the respective species abundance. Metatranscriptomic reads were then simulated using the RSEM simulator (Li and Dewey, 2011).

We also exploited the Mock16 dataset containing real sequencing data obtained from a synthetic mix of 16 different bacteria (Ternus et al., 2021; Table 1). Finally, to test MetaGT in a real-life environment we used sequencing data from the human and snail gut microbiomes: HumanGut (Lloyd-Price et al., 2019) and SnailGut datasets (Yang et al., 2022).

### Quality evaluation

To evaluate assembled sequences we aligned them against the true set of transcripts with minimap2 (Li, 2018). These alignments are processed to estimate the number of unmapped contigs and percentage of captured transcripts. A reference transcript is considered to be captured if 95% of its bases are covered by a single alignment. For simulated data the ground truth set contained only transcripts with expression level TPM ≥ 1. For Mock16 we used the entire reference set as the true expression is unknown.

Further, to access assembly correctness we estimated the number of misassemblies using rnaQUAST (Bushmanova et al., 2016) and simply by mapping assembled transcripts to the reference genomes with minimap2. A contig is considered as misassembled if its parts (i) either align to different genomes, or (ii) map to the same genome at least 1 kbp apart (Gurevich et al., 2013).

For real sequencing data we also estimate assembly completeness using predicted coding regions in genomic contigs. We similarly map resulting transcripts to the predicted CDS using minimap2, and for each CDS we compute its fraction captured by a single transcript alignment.

## Results

Table 2 demonstrates that on simulated data simultaneous use of metagenomic and metatranscriptomic data allows to reduce the total number of assembled sequences by 12% and the amount of unaligned contigs by 15% on average compared to assemblies obtained solely from RNA-Seq data. Importantly, MetaGT shows a 16% average increase in the number of captured reference transcripts. Moreover, the difference appears to be more significant for complex communities, where homologous genes are frequent and *de novo* assembly becomes challenging: for Complex32 simulated dataset MetaGT restored 25% more complete RNA sequences compared to rnaSPAdes. With respect to assembly correctness, both rnaSPAdes and MetaGT show a rather low number of misassembled contigs according to rnaQUAST. However, MetaGT is being somewhat less accurate on Medium20 and Complex35 datasets according to the genomic approach to misassembly detection (see “Materials and methods”).

**Table 2:** Assembly results on simulated data and synthetic community.

Dataset	Tool	# Sequences assembled	# Unaligned sequences	Captured transcripts (%)	Misassemblies (rnaQUAST)	Misassemblies (genomic)
Simple7	rnaSPAdes	16,132	1,670	75.8	**0**	7
	MetaGT	15,511	**1,628**	**79.0**	**0**	**5**
Medium20	rnaSPAdes	51,736	11,360	50.4	**0**	**2**
	MetaGT	45,620	**9,999**	**59.4**	**0**	7
Complex32	rnaSPAdes	90,883	8,656	50.1	2	**5**
	MetaGT	72,148	**5,950**	**58.7**	**0**	18
Mock16	rnaSPAdes	18,916	2,077	4.2	118	194
	MetaGT	6,364	**462**	**9.3**	**0**	**1**
	MetaGT + reference	13,711	544	**9.6**	4	20

Comparison between metatranscriptome assemblies obtained with rnaSPAdes and MetaGT pipeline on three simulated and 1 Mock datasets. The ground truth set contains 16,884 expressed transcripts (simulated TPM > 1) for the Simple7 sample, 48,752 for Medium20 and 81,924 for Complex32. For Mock16 the entire set of reference transcripts was used as the ground truth. Assembled sequences were mapped to the respective expressed reference transcripts with minimap2. A reference transcript is considered as captured if it is covered by a single assembled sequence by at least 95% of its length. The best values are highlighted with bold.

Importantly, on the synthetic Mock16 dataset MetaGT yields a more significant improvement: a 4-fold drop in unaligned contigs and almost a double increase in captured reference transcripts. Moreover, Table 2 shows that MetaGT eliminated almost all misassembled contigs from the initial rnaSPAdes assembly (0 vs. 118 according to rnaQUAST).

Since in real-life metagenomic projects researchers obtain metagenome-assembled genomes (MAGs), we also tested MetaGT on Mock16 data by providing reference genomes instead of raw genomic reads. Interestingly, using the genome assembly of a significantly better quality results only in a marginal improvement in transcriptome assembly. As per Table 2, this approach allowed to capture only 121 additional transcripts (0.3%) and resulted in a few more misassemblies compared to the original read-based approach. However, we predict that for more complex bacterial communities exploiting MAGs instead of draft genome assembly could potentially lead to a more noticeable improvement.

**Figure 3:**
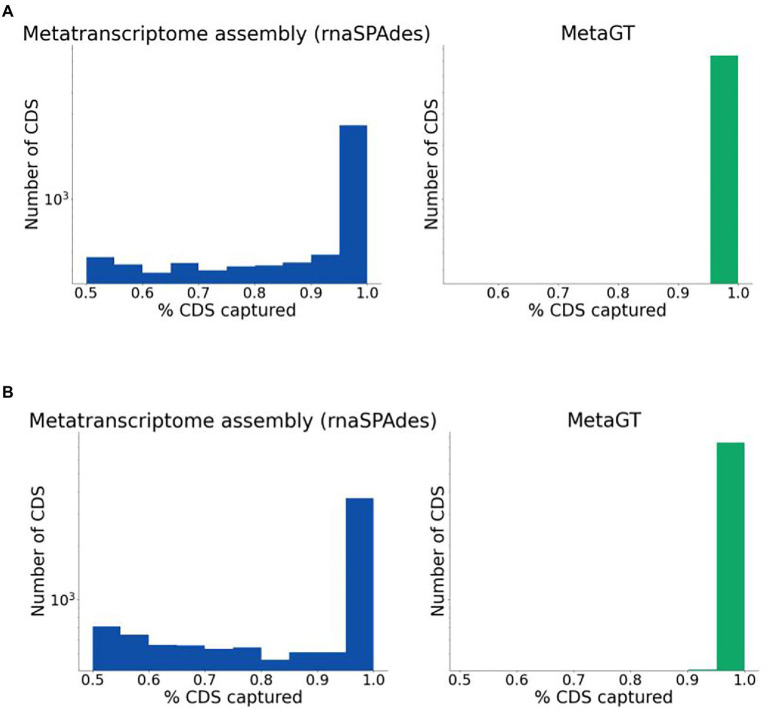
Completeness of sequences assembled from real data. **(A)** Histogram for percentage of coding regions captured by rnaSPAdes (left) and MetaGT (right) assemblies on the Mock16 dataset. The histogram is shown in logarithmic scale. **(B)** Same as **(A)**, but for the HumanGut dataset.

For real sequencing data (Mock16 and HumanGut) we estimated completeness of assembled transcripts using predicted genes as described in the Quality evaluation section. Expectedly, combined usage of metatranscriptomic and metagenomic data yields a significantly higher percentage of fully captured transcripts on both datasets (Figures 3A,B respectively). On Mock16 MetaGT reconstructs 6,425 full-length transcripts (95% of bases captured), which is 2.5-fold more than assembled by rnaSPAdes (2,596). Similarly, for real HumanGut dataset MetaGT reports 2-fold more complete transcripts compared to rnaSPAdes (7,465 vs. 3,649), thus proving that the developed pipeline is efficient on real data and allows to significantly reduce fragmentation of the assembly. It is important to point out that this striking improvement also demonstrates that real sequencing data is noticeably more challenging for *de novo* transcriptome assembly compared to simulated data.

**Figure 4:**
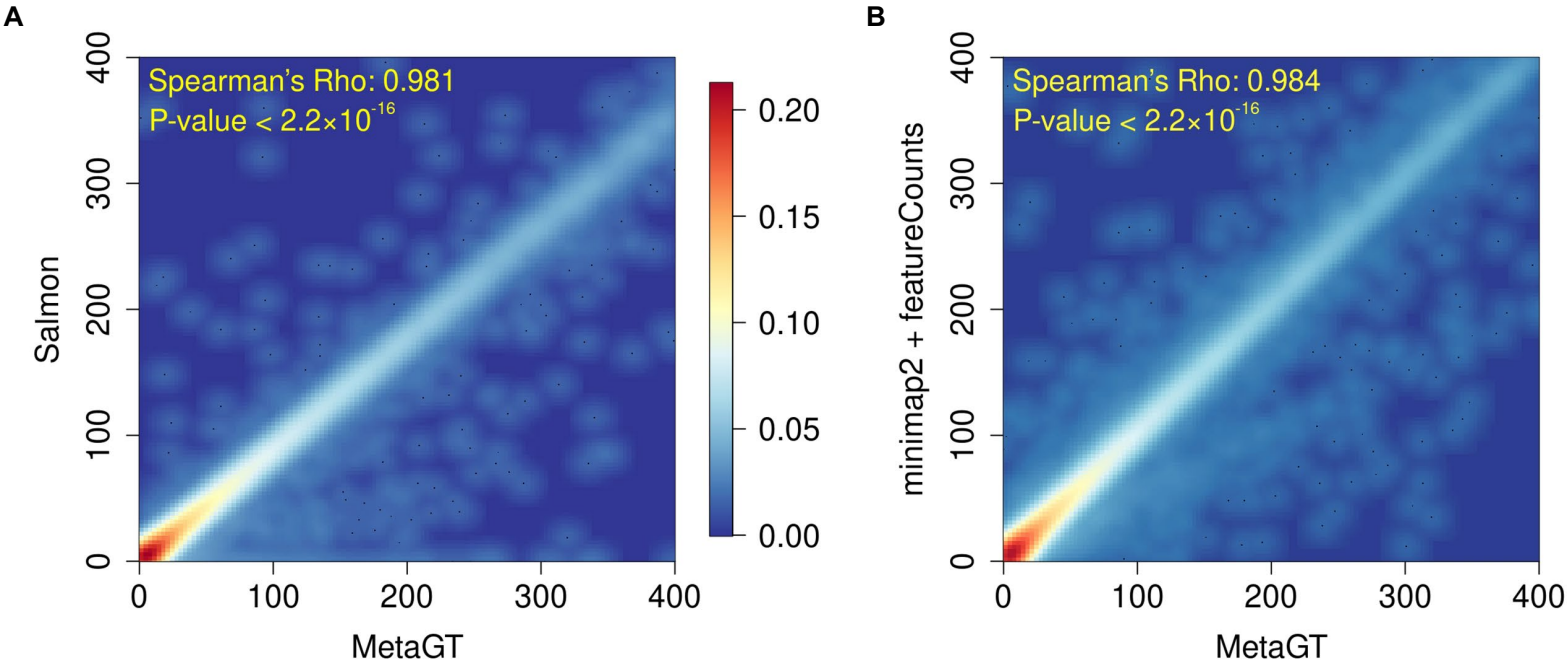
Heatmaps showing comparison between gene counts obtained with MetaGT and **(A)** approach used in the original study (Prodigal and Salmon), and **(B)** alignment based approach that involves Prodigal, mimimap2 and featureCounts.

To assess MetaGT ability to estimate gene expression levels, we reproduced quantification results from a recent metatranscriptomic study of a deep-sea snail gut microbiome (Yang et al., 2022). Abundances generated with the MetaGT pipeline were compared against the approach exploited in the original work, in which Salmon (Patro et al., 2017) was used to quantify genes predicted by Prodigal (Hyatt et al., 2010). In addition, we also computed expression levels for the same set of genes with an alignment-based approach by using minimap2 (Li, 2018) and featureCounts from the Subread package (Liao et al., 2014). We then estimated similarity between counts provided by MetaGT and two other approaches by computing Spearman’s rank correlation coefficient (Figures 4A,B respectively). In both cases the results appear to be highly similar with Spearman’s Rho > 0.98 (*p*-values < 2.2 × 10^−16^), which suggests that MetaGT provides meaningful quantification results as well.

## Discussion

While combining metatranscriptomic data with metagenome assemblies obtained from the same sample seems to be intuitive, to the best of our knowledge no modern bioinformatics software implements such an idea, with a solo exception of TAG assembler, support of which has been unfortunately discontinued a long time ago. As described previously, TAG maps RNA-Seq reads using Bowtie2 (Langmead and Salzberg, 2012) and k-mer matching to the metagenomic de Bruijn graph, and further derives transcript sequences from the corresponding alignment paths in the graph. In comparison to MetaGT, the approach implemented in TAG can be useful for reconstructing transcripts with extremely low coverage, i.e., when the number of reads is insufficient for *de novo* transcriptome assembly. At the same time, TAG may output incomplete and fragmented transcripts when RNA-Seq reads do not cover the entire coding region. In contrast, MetaGT exploits predicted genes to fill in the gaps and restore complete transcripts sequences.

In this work we present a pipeline that performs *de novo* assembly of metagenome and metatranscriptome sequencing data using existing software and combines the results in order to reconstruct and further quantify full-length transcripts. Providing complete coding sequences as the result of the assembly pipeline may significantly improve quality of the downstream analysis, such as functional annotation, gene ontology and differential expression analysis. In the view of growing popularity of metatranscriptomic sequencing we believe that MetaGT will be a useful instrument in the field and will allow researchers to perform high-quality studies without spending time developing custom in-house pipelines.

## Data availability statement

The datasets analyzed in this study can be found in the NCBI short read archive (SRA) under accession numbers SRR5947833, SRR5947907, SRR10175815, SRR10175826, SRR8397925, SRR8416101. Simulated data is published on Zenodo with DOI: 10.5281/zenodo.7152149. The pipeline is implemented in NextFlow and is freely available at https://github.com/ablab/metaGT.

## Author contributions

DS designed and developed MetaGT software. DS and VK tested the software. DS, AK, and AP wrote the manuscript. RF and VK provided the data for testing. AK and AL acquired funding. RF, AK, and AP managed the project and supervised the research. All authors contributed to the article and approved the submitted version.

## Funding

The research was carried out in part by computational resources provided by the Resource Center “Computer Center of SPbU.” The reported study was funded by the Russian Scientific Foundation, project number 19-14-00172.

## Conflict of interest

The authors declare that the research was conducted in the absence of any commercial or financial relationships that could be construed as a potential conflict of interest.

## Publisher’s note

All claims expressed in this article are solely those of the authors and do not necessarily represent those of their affiliated organizations, or those of the publisher, the editors and the reviewers. Any product that may be evaluated in this article, or claim that may be made by its manufacturer, is not guaranteed or endorsed by the publisher.

## Footnotes

^1^ https://github.com/TransDecoder/TransDecoder

^2^ https://github.com/TransDecoder/TransDecoder
